# Supplementary material for: Understanding of Final Year Medical, Pharmacy and Nursing Students in Pakistan towards Antibiotic Use, Antimicrobial Resistance and Stewardship: Findings and Implications
Source: Antibiotics (Basel). 2023 Jan 10;12(1):135. doi: 10.3390/antibiotics12010135 (PMC9854661; doi:10.3390/antibiotics12010135)
Supplement: Supplementary file 1 [file antibiotics-12-00135-s001.zip › antibiotics-2060201-supplementary.pdf]

Supplementary Figures and Tables

Figure S1: Sources of information (*n*=1251)

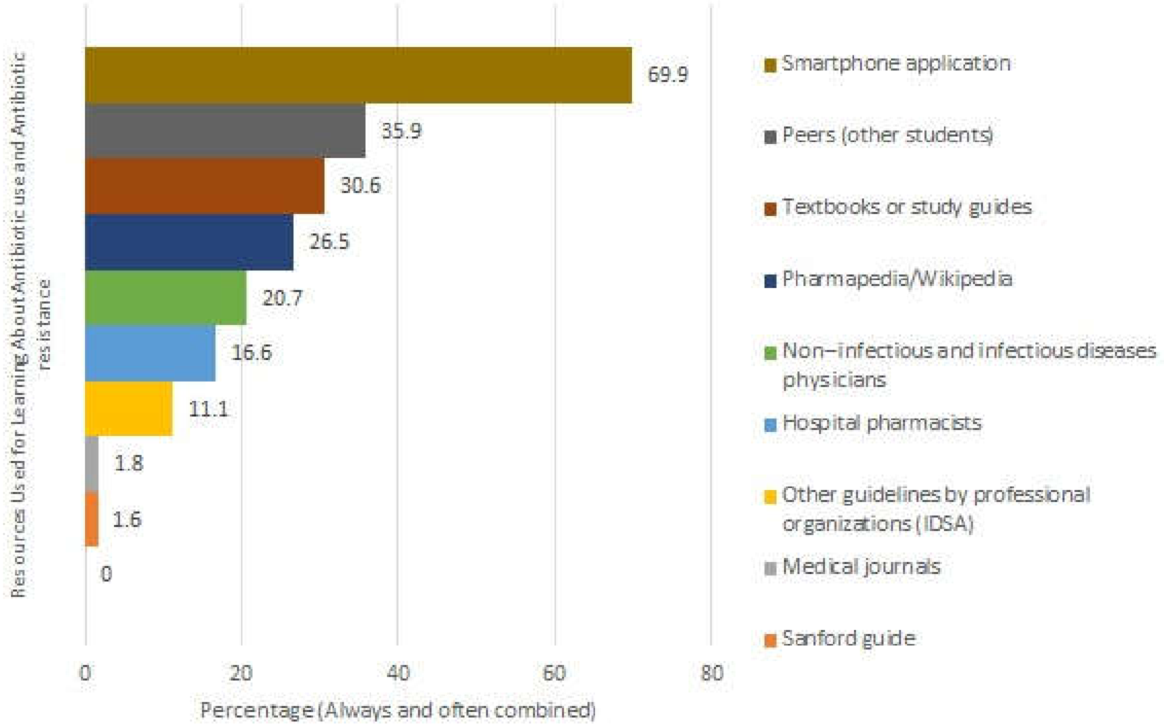

Table S1: Demographic characteristics of study participants (n=1251)

Variables	Frequency (n)	Percentage (%)
**Sex**		
Female	916	73.2
Male	335	26.8
**Age (years)**		
<20	14	1.1
20-25	1105	88.3
>25	132	10.6
**Family income (PKR)**		
<25000	32	2.6
25000-75000	639	51.1
>75000	579	46.3
**Student type**		
Medical	360	28.8
Pharmacy	471	37.6
Nursing	420	33.6
**Institute type**		
Public	913	73.0
Private	338	27.0
**Residence**		
Rural	752	60.1
Urban	499	39.9
**Parents profession**		
Medical	237	18.9
Non-Medical	1014	81.1
**ASP Training**		
Yes	369	29.5
No	882	70.5
**Antibiotic use in last 6 months**		
Yes	431	34.5
No	820	65.5

NB: ASP: Antimicrobial Stewardship, (PKR) = Pakistani Rupee

Table S2: Resources Used for Learning About Antibiotic use and Antibiotic resistance

S. No	Questions	Always (%)	Often (%)	Sometimes (%)	Seldom (%)	Never (%)
1	Textbooks or study guides	42 (3.4)	340 (27.2)	378 (30.2)	348 (27.8)	143 (11.4)
2	Smartphone application	227 (18.1)	648 (51.8)	314 (25.1)	62 (5.0)	0 (0.0)
3	Hospital pharmacists	2 (0.2)	205 (16.4)	222 (17.7)	329 (26.3)	493 (39.4)
4	Non–infectious and infectious diseases physicians	18 (1.4)	241 (19.3)	414 (33.1)	248 (19.8)	330 (26.4)
5	Medical journals	0 (0.0)	22 (1.8)	105 (8.4)	402 (32.1)	722 (57.7)
6	Peers (other students)	33 (2.6)	416 (33.3)	579 (46.3)	208 (16.6)	15 (1.2)
7	Sanford guide	3 (0.2)	18 (1.4)	139 (11.1)	1091 (87.2)	0 (0.0)
8	Infectious Diseases Society of America guidelines	0 (0.0)	0 (0.0)	4 (0.3)	94 (7.5)	1153 (92.2)
9	Other guidelines by professional organizations	2 (0.2)	136 (10.9)	437 (34.9)	319 (25.5)	357 (28.5)
10	Pharmapedia/Wikipedia	45 (3.6)	287 (22.9)	504 (40.3)	368 (29.4)	47 (3.8)

Table S3: Details of Medical, Pharmacy and Nursing Institutions

**Name of Institute**	**Type of institute**	**Sector of institute**	**Frequency (*n*)**	**Percentage (%)**
Rehman College Of Nursing & Allied Health Sciences, Khanewal	Nursing	Private	73	5.8
Rehman College Of Nursing & Allied Health Sciences, Mianwali	Nursing	Private	72	5.8
Rehman College Of Nursing & Allied Health Sciences, Multan	Nursing	Private	60	4.8
Rehman College Of Nursing & Allied Health Sciences, Vehari	Nursing	Private	46	3.7
Bahawal Victoria Hospital, Bahawalpur	Medical	Public	60	4.8
Faculty of Pharmacy, Bahauddin Zakariya University, Multan	Pharmacy	Public	49	3.9
College of nursing, Bahawal Victoria Hospital, Bahawalpur	Nursing	Public	65	5.2
Fatima Medical University, Faisalabad	Medical	Public	65	5.2
Faculty of pharmacy, Islamia University, Bahawalpur	Pharmacy	Public	64	5.1
Institute of Pharmacy, Faculty of Pharmaceutical and Allied Health Sciences, Lahore College for Women University, Lahore 54000, Pakistan	Pharmacy	Public	46	3.7
Lady Willingdon Hospital School of Nursing, Lahore	Nursing	Public	66	5.3
School Of Nursing, Allied Hospital, Faisalabad	Nursing	Public	38	3.0
Nishtar Medical University, Multan	Medical	Public	39	3.1
Punjab University, College of pharmacy, University of the Punjab, Lahore	Pharmacy	Public	115	9.2
Department of Pharmacy, Quaid e Azam University, Islamabad.	Pharmacy	Public	48	3.8
Rawalpindi Medical University, Rawalpindi	Medical	Public	41	3.3
Services Institute of Medical Sciences, Lahore	Medical	Public	51	4.1
Sharif Medical & Dental College, Lahore	Medical	private	26	2.1
Shalimar medical and dental college, Lahore	Medical	private	32	2.6
Sahiwal Medical college, Sahiwal	Medical	Public	37	3.0
Department of Pharmacy, The University of Lahore	Pharmacy	Public	55	4.4
Faculty of Pharmacy, University of Sargodha, Sargodha	Pharmacy	Public	68	5.4
